# PATHWAYS LINKING ICT UTILIZATION TO CHRONIC DISEASES SELF-MANAGEMENT AMONG OLDER ADULTS IN CHINA

**DOI:** 10.1093/geroni/igae098.3682

**Published:** 2024-12-31

**Authors:** Qingru Chen, Yan-Yan Chen, Ke Gong, Xuefeng Zhang, Lirong Zhao

**Affiliations:** Huadong Hospital Affiliated to Fudan University, Shanghai, Shanghai, China (People’s Republic); Fudan University, Shanghai, Shanghai, China (People’s Republic); Huadong Hospital affiliated to Fudan University, Huadong Hospital Affiliated to Fudan University, Shanghai, China (People’s Republic); Huadong Hospital affiliated to Fudan University, Shanghai, Shanghai, China (People’s Republic); Huadong Hospital affiliated to Fudan University, Shanghai, Shanghai, China (People’s Republic)

## Abstract

The underlying mechanisms linking information and communication technology (ICT) utilization to self-management are currently unclear. This cross-sectional study aims to examine the mediating role of health literacy, self-efficacy, and social support in the relationship between ICT utilization and chronic disease self-management among older adults in China. A total of 590 older adults (≥60 years) with at least one chronic disease were recruited from a tertiary hospital in Shanghai between July 2023 and June 2024. Questionnaire data were recorded on self-reported socio-demographics, self-management, social support, health literacy, self-efficacy, ICT utilization. Pearson’s correlation analysis identified the relationship between these factors and self-management. Serial multiple mediation model was used to test the hypothesized relationships. The results show ICT utilization, health literacy, self-efficacy, social support, and chronic disease self-management were found significantly correlated (p< 0.001). ICT utilization not only has a direct positive impact (effect=0.2377; 95%CI=0.1826-0.2927) on chronic disease self-management, but also has an indirect impact on chronic disease self-management through the independent mediating role of health literacy (effect=0.0912; 95%CI=0.0608-0.1240) and social support (effect=0.0186; 95%CI=0.0048-0.0367) and the chain mediating role of health literacy and self-efficacy (effect=0.0223; 95%CI=0.0128-0.0342), the chain mediating role of health literacy and social support (effect=0.0104; 95%CI=0.0044-0.0187), the chain mediating role of health literacy, self-efficacy, and social support (effect=0.0020; 95%CI=0.0008-0.0036).These findings suggest that ICT utilization can enhance chronic disease self-management among older adults and indirectly affects it through increasing health literacy, self-efficacy while providing more social support.

